# Case of Mr. Dyce Sombre. (*Morning Chronicle.*)

**Published:** 1851-01-01

**Authors:** 


### CASE OF MR. DYCE SOMBRE.

(*Morning Chronicle.*)

On Friday, the 13th of December, this anomalous, interesting, and perplexing case of lunacy was again brought before the Court of Chancery. In the course of the argument on the petition of Mrs. Dyce Sombre for permission to sell out 10,000*l.*, part of the settled stock, in order to pay certain calls upon railway shares, Mr. Bethell prayed that the consideration of the matter might stand over, "as there was great difficulty in ascertaining where the unfortunate lunatic now was. He had not been heard of for eight weeks, and it was a matter of doubt whether he was dead or alive." This indeed would be a sad termination to this expensively-litigated case, should Mr. Bethell's surmise turn out to be the fact. But, we ask, what precautions have been taken to prevent such a result? Mr. Dyce Sombre, after a protracted and contested inquiry, was found, by inquisition, of unsound mind, and incapable of managing himself and his affairs. After being for a short period under strict surveillance, the lord chancellor thought it expedient to grant him permission to go abroad, having at his own immediate command an income of at least 10,000*l.* per annum. This course of procedure struck every person accustomed to the judicial management of these cases as extraordinary and anomalous. Mr. Dyce Sombre was either a lunatic, requiring to be placed under surveillance, or he was of sane mind, and competent to take care of himself and property. It was asserted by all those who supported the inquisition, that Mr. Dyce Sombre was not only a lunatic, but a dangerous lunatic; it was sworn in evidence, that Mrs. Dyce Sombre's life had repeatedly been in danger, and that he had, under the overpowering influence of several delusions, threatened violence to other parties; in fact, that he was quite unfit to be at large. Without the slightest evidence that these alleged dangerous tendencies had subsided, the chancellor removed all restraint and surveillance, and allowed Mr. Sombre to reside in England, France, Russia, and Poland, allowing him the uncontrolled income of 10,000*l.* a year. For some years Mr. Dyce Sombre has made the capital of France his home, and in that city we presume he has so mysteriously disappeared. The question now arises whether those officially connected with the case were justified in allowing this alleged dangerous lunatic to be at large in Paris, having at command so large an income.

For the last five or six years various attempts have been made by the lunatic to supersede the commission. He has, we believe, been examined by at least fifty different men of eminence in England, France, and Germany. Many have certified to his continued lunacy, and others have sworn that he is perfectly sane, and competent to have the management of himself and his affairs. In this country the following physicians have made affidavits of Mr. Sombre's lunacy—Dr. Conolly, Sir J. Clark, Bart., Dr. Soulby, Dr. Bright, Dr. Sutherland, and, we believe, Dr. Monro. On the opposite side we find the names of Dr. Paris, Dr. Mayo, Dr. Copeland, and several other men of eminence. As there appears to be almost an equal balance of opinion among the English faculty, it was proposed that three medical men of reputation in matters of lunacy should be selected, who had given no opinion of the case, with the view to an impartial inquiry, and final settlement of the matter. After much discussion and disputation, Dr. Seymour, who has had considerable experience in lunacy cases, having acted for ten or fifteen years as one of the metropolitan commissioners in lunacy; Dr. Forbes Winslow, also a gentleman of considerable practice in cases of insanity, and Mr. Lawrence, the celebrated surgeon of Bethlem Hospital, were nominated, with the approbation of Mr. Dyce Sombre and his legal advisers, to constitute the court of inquiry. The gentlemen previously mentioned consented to go fully into the case, but declined doing so without having in the first instance the consent of the lord chancellor, then Lord Cottenham, to the proposed investigation. They considered, and that justly and properly, that as Mr. Sombre had been pronounced a lunatic, and was still under the protection of the Court of Chancery, they could not entertain the question of Mr. Sombre's sanity or insanity, without interfering with the jurisdiction of the lord chancellor. This matter was placed in its proper light before Mr. Sombre and his distinguished advocate, Mr. Rolt, but unfortunately an application to the lord chancellor was not considered necessary as a preliminary step to the inquiry, and consequently the matter dropped. Subsequently to this, Drs. Paris, Mayo, and Copeland, made affidavits in Mr. Dyce Sombre's favour. They were brought before the Chancellor, and the petition based upon them was dismissed, the Court of Chancery being of opinion that Mr. Dyce Sombre continued of unsound mind. We believe it was afterwards arranged by Lord Cottenham that Dr. Seymour and Dr. Winslow, conjointly with Mr. Lawrence, should investigate the matter; but the Lord Chancellor's severe indisposition, and subsequent resignation of the Great Seal, interfered unfortunately with this arrangement, which it was hoped would finally, at least for a period, put an end to this expensive, anxious, and protracted suit. Should Mr. Sombre be still alive, it will become a serious question for the lord chancellor to consider, whether it is not a sad reflection on the proceedings of his court that so anomalous a case should any longer be permitted to exist. If Mr. Dyce Sombre be a lunatic, and dangerous to society and himself in consequence of his delusions, it may be asked, is Paris the proper place for him to live in, when it is generally known that he has large sums of money at his command? Again, if he be a lunatic, why should the chancellor make an exception in his case, and permit him not only to be at large, under no kind of surveillance, but to reside out of the jurisdiction of his court? If he be competent to spend safely and sanely an income of 10,000*l.* a year, and have his unfettered liberty, what reason can be urged in opposition to a *supersedeas* of the commission of lunacy? Mr. Dyce Sombre and his friends have just reason to complain of his present position; he either should be considered and treated as insane or as sane: there is no intermediate stage recognisable in law between lunacy and sanity which brings the person within the jurisdiction of the Court of Chancery. A serious amount of responsibility rests upon those who in the slightest degree countenance the present anomalous position of Mr. Dyce Sombre. Should it turn out that his insanity has been taken advantage of, and that his life has been sacrificed, who would be the parties that would be censured? Paris is the last city in which a chancery lunatic, particularly with his mind, as is alleged, filled with dangerous delusions, should be set at large. A sane man, with all his wits about him, would, with an income of 10,000*l.* per annum, find some difficulty in steering clear of the temptations that so constantly beset the path of those resident in that fascinating city. We would, without giving an opinion of Mr. Sombre's state of mind, recommend all parties connected with the case to look well into the matter, with the view of bringing the question to a satisfactory issue. For the credit of the Court of Chancery, we sincerely hope this matter will meet with prompt and careful consideration.

